# Short Meditation Trainings Enhance Non-REM Sleep Low-Frequency Oscillations

**DOI:** 10.1371/journal.pone.0148961

**Published:** 2016-02-22

**Authors:** Daniela Dentico, Fabio Ferrarelli, Brady A. Riedner, Richard Smith, Corinna Zennig, Antoine Lutz, Giulio Tononi, Richard J. Davidson

**Affiliations:** 1 Department of Psychiatry, University of Wisconsin Madison, 6001 Research Park Blvd, Madison, WI, 53719, United States of America; 2 Waisman Center for Brain Imaging and Behavior, University of Wisconsin Madison, Madison, WI, 53705, United States of America; 3 Center for Healthy Minds, University of Wisconsin Madison, Madison, WI, 53705, United States of America; 4 Lyon Neuroscience Research Center, INSERM U1028, CNRS UMR5292, Lyon 1 University, Lyon, 69500, France; 5 Department of Psychology, University of Wisconsin Madison, Madison, WI, 53706, United States of America; University of Oxford, UNITED KINGDOM

## Abstract

**Study Objectives:**

We have recently shown higher parietal-occipital EEG gamma activity during sleep in long-term meditators compared to meditation-naive individuals. This gamma increase was specific for NREM sleep, was present throughout the entire night and correlated with meditation expertise, thus suggesting underlying long-lasting neuroplastic changes induced through prolonged training. The aim of this study was to explore the neuroplastic changes acutely induced by 2 intensive days of different meditation practices in the same group of practitioners. We also repeated baseline recordings in a meditation-naive cohort to account for time effects on sleep EEG activity.

**Design:**

High-density EEG recordings of human brain activity were acquired over the course of whole sleep nights following intervention.

**Setting:**

Sound-attenuated sleep research room.

**Patients or Participants:**

Twenty-four long-term meditators and twenty-four meditation-naïve controls.

**Interventions:**

Two 8-h sessions of either a mindfulness-based meditation or a form of meditation designed to cultivate compassion and loving kindness, hereafter referred to as compassion meditation.

**Measurements and Results:**

We found an increase in EEG low-frequency oscillatory activities (1–12 Hz, centered around 7–8 Hz) over prefrontal and left parietal electrodes across whole night NREM cycles. This power increase peaked early in the night and extended during the third cycle to high-frequencies up to the gamma range (25–40 Hz). There was no difference in sleep EEG activity between meditation styles in long-term meditators nor in the meditation naïve group across different time points. Furthermore, the prefrontal-parietal changes were dependent on meditation life experience.

**Conclusions:**

This low-frequency prefrontal-parietal activation likely reflects acute, meditation-related plastic changes occurring during wakefulness, and may underlie a top-down regulation from frontal and anterior parietal areas to the posterior parietal and occipital regions showing chronic, long-lasting plastic changes in long-term meditators.

## Introduction

Increasing evidence indicates that meditation practices induce long-term neuronal plastic changes, as reflected by an increase in central and posterior EEG gamma power. Berkovich-Ohana and coworkers related this EEG feature to enhanced attentional skills and sensory awareness [1]. Lutz and coworkers showed that the intensity of the gamma changes during a compassion meditation correlated with the duration of training in long-term practitioners, and remained higher in the post meditation baseline [2]. A parietal-occipital increase in gamma power has been reported during Vipassana meditation, a style of mindfulness-based meditation [3]. Furthermore, our group has recently shown in experienced practitioners a meditation-induced increase in parietal-occipital EEG gamma activity during whole night NREM sleep [4]. This increase was correlated with lifetime daily practice, clearly suggesting that meditation training is able to induce local traces in sleep EEG, and that long-term meditation experience is required for building-up this effect. The finding of a gamma increase during NREM sleep, a stage characterized by low muscle tone, is particularly relevant considering that spontaneous EEG gamma power in waking is contaminated with electromyogenic artefactual sources [5] and, together with the direct assessment of the absence of group differences in the electromyographic and electrooculographic derivations [4], appears to confirm the cortical origin of this activity. Additionally, the increase in gamma activity appears to be rather a marker of duration of training than a marker of a specific practice, given it has been found to characterize experienced meditators in different styles, either focusing more on cognitive (mindfulness meditation [1,3]) or emotional regulatory strategies (compassion meditation [2]).

The aim of the present study was to investigate the acute effects of meditation practice, as they may be revealed by measuring EEG activity during sleep. Our prediction was that an intensive meditation session would result in sleep increase in low frequency activity (1–12Hz) in long-term meditators. The main line of evidence supporting this hypothesis comes from studies assessing the specific effects on the brain of different forms of training. It has been demonstrated that motor and mental trainings are able to induce task-related neuroplastic changes, in waking as well as during subsequent sleep [6–8]. Experience—dependent plasticity during sleep can be measured as changes in power in low-frequency bands, ranging from the slow-wave (1–4 Hz) to the alpha range (8–12Hz). For instance, region-specific effects in the 1–11 Hz frequency band can be induced during sleep by different learning tasks such as an intense and prolonged visuomotor or language task during the preceding wakefulness [7]. Our primary hypothesis is that an increase in a wide range of low-frequency activities during sleep, as a consequence of learning during wakefulness, reflects process-specific aspects of the learning experience [9]. Conversely the task-specificity will be reflected in the specific localization of the effect.

In this study we asked the same population of experienced practitioners who underwent baseline sleep recordings to intensely meditate for 8 hours in two different sessions, dedicated to the Vipassana and the Metta style of practice respectively. Vipassana meditation is a style of mindfulness or open monitoring practice [10]. It involves practicing non-judgmental open monitoring of the spontaneous flow of conscious experience to gain insight about the nature of mental patterns. It aims to cultivate a less reactive awareness to emotions, thoughts and sensations occurring in the present moment to prevent them from being automatically amplified and creating mental distress. It is usually preceded by focused attention on an intended object with the aim to tame the mind that is usually unstable, disorderly, and occupied by a stream of barely perceived inner chatter. Metta meditation sustains the focus of attention on a specific individual or a group of individuals while cultivating feeling of benevolence and loving-kindness for others, accompanied by aspiration such as: “May all beings find happiness and the causes of happiness, and be free from suffering and the causes of suffering.” [9–11]. As both meditations are thought to recruit executive, attention-based functions, we predict that the increase in low frequency activity during sleep will recruit overlapping regions important for cognitive control and we predict that we will find a common and spatially specific pattern of activation as a result of the training [2,12,13]. Moreover, given recent work revealed domain-specific plastic changes in neural activity induced by different meditation practices [14], we will also explore the possibility of region-specific activation for each meditation style. We recorded whole-night high-density EEG following each of these sessions and compared this activity with the whole night baseline recordings. We also investigated how these changes were predicted by meditation life experience and how they related to changes in behavioral attentional and emotional measures.

## Materials and Methods

### Participants

A population of twenty-nine right-handed long-term meditators (mean age = 50.7 ± 10.4, 15 female) was initially recruited based on a history of daily meditation practice of at least 3 years and the participation to a minimum of 3 one-week intensive retreats. Mean duration of meditation training was 15.6 years (± 7.8, SD). All participants were experienced in attentional and emotional styles of meditation practices, as taught within the framework of Theravada and Tibetan Buddhist traditions. From the perspective of the attentional regulatory strategies involved, the complexity and heterogeneity of these meditation practices can be related back to two main styles: focused attention (FA) and open monitoring (OM) [10]. FA meditation involves sustaining the attention on a specific object, like the breath. During OM meditation, in contrast, the spontaneous flow of cognitive and emotional patterns is monitored in a non-judgmental fashion [10]. FA and OM represent the core training practices of Vipassana or *insight* meditation. It has been pointed out that training self-observation not only improves cognitive, but also enhances the emotional regulatory abilities, by decreasing emotional reactivity and by monitoring the emotional tone of the subjective experience [10]. However, there are practices that more specifically target emotion regulation and self-related processes. These practices belong to the Metta or *loving-kindness* (LK) tradition. Metta meditation specifically trains loving-kindness, or the wish of happiness towards ourselves and others, as well as compassion, the wish for all beings to be free from suffering [15]. The long-term meditators underwent three whole night sleep hd-EEG recordings, baseline, post a full day of Vipassana practice, as well as post a full day of Metta meditation practice. Two participants did not complete the Vipassana meditation day of practice and 3 participants were excluded from the analyses due to sleep disturbances (see below).

We also enrolled a group of 38 age and gender matched control individuals, from which the 24 best matches for the long-term meditators were chosen (mean age = 48.47 ± 10.3, 13 female). The meditation naïve individuals did not undergo 2 intensive days of practice, but they were utilized as a control group to assess for potential, nonspecific effects of time on sleep architecture and EEG activity.

After an initial phone screening to collect the medical and psychiatric history, each study participant underwent a thorough in-person screening, which included several questionnaires (see below). Sleep-disordered breathing and sleep-related movement disorders were also established/excluded with in-laboratory polysomnography (see below). All participants provided written informed consent and were instructed to maintain regular sleep-wake schedules in the week preceding EEG recordings. This study was approved by the Institutional Review Board of the University of Wisconsin-Madison.

### Study design

The experimental design was structured in 3 sessions, a baseline session, and two days of practice of Vipassana and Metta meditations, respectively. The order of the days of meditation was randomized across participants. The 3 sessions were separated by a time interval of 160.8 ± 38.0 days. Participants arrived at the sleep laboratory 5 hours before their usual bedtime for EEG set-up and waking recordings. The waking session was comprised of an auditory Go/No-Go attentional task and an assessment of positive and negative affects which was performed before and after the EEG recordings by means of the PANAS-now questionnaire, a version of the PANAS scale that is sensitive to day by day fluctuations of the emotional state [16]. These cognitive and emotional evaluations will be discussed elsewhere. Within one hour of their usual bedtime, participants were allowed to sleep undisturbed in the laboratory, after completing a Stanford Sleepiness Scale [17]. Additional measures were collected the next day.

The two days of practice closely reproduced the structure of a meditation retreat. Six 45-min sessions of sitting meditation, three in the morning, three in the afternoon, were separated by four 30-min sessions of walking meditation. The first 45-min sitting meditation session began at 8:15 am with a short guided meditation. The morning and afternoon sessions were separated by a 1-h lunch break, followed by a 30-min dharma talk.

The order of Vipassana and Metta meditation sessions was randomized across participants. Two subjects didn't complete the Vipassana meditation session, and their data were excluded from the analyses. For the control participants, no intervention was performed.

Sleep EEG recordings with a 256 channel hdEEG system (Electrical Geodesics Inc., Eugene, OR), were collected for each of those three visits.

### Self-reported measures

The socioeconomic status [18] was administered to assess the level of education. The presence of depression and other mental health issues was assessed through the Quick Inventory of Depressive Symptoms (QIDS) [19] and the Symptom Checklist-90-Revised (SCL-90) [20] and a score >2 represented an exclusion criterion for each of the two questionnaires. Symptoms of common sleep disorders, such as restless leg syndrome and obstructive sleep apnea were assessed using validated sleep rating scales, including the Insomnia Severity Index (ISI) [21], the Fatigue Severity Scale (FSS) [22], the Epworth Sleepiness Scale [23], a sleep history questionnaire, and the Stanford Sleepiness Scale [17]. Cut-off for exclusion were an ISI >10 and/or an FSS >4 and/or an ESS >9.

### Meditation practice

Participants’ reports of their average hours of formal (sitting and walking) meditation training per week allowed us to assess the total amount of lifetime hours of practice (on average 8,762, ranging from 1,526 to 32,349 hours), as well as to derive the specific contribution of meditation styles to their training in terms of percentage over the total.

### Sleep PSG assessment and hdEEG data analysis

Six mastoid-referenced channels, which included F3, F4, C3, C4, O1, and O2, together with a sub-mental electromyogram and an electrooculogram were utilized to perform sleep staging, in 30-second epochs according to standard criteria [24] using Alice^®^ Sleepware (Philips Respironics, Murrysville, PA). Sleep EEG recordings were scored by a registered polysomnographic technologist who was blind to the experimental conditions. PSG recordings during the baseline session were reviewed by a board certified sleep medicine physician. Individuals with sleep disorders, which included sleep-related movement disorders (periodic limb movement arousal index >10 / h, 2 participants), and sleep-disordered breathing (apnea—hypopnea index >10 ⁄ h, 1 participant), were excluded from the analyses. All-night sleep hdEEG recordings were collected at a sampling frequency of 500 Hz with vertex-referencing, using a NetAmps 300 amplifier and NetStation software (Electrical Geodesics Inc., Eugene, OR). After applying a first-order high-pass filter (Kaiser type, 0.1 Hz) to eliminate the DC shift, data were band-pass filtered (1–50 Hz), and down-sampled to 128 Hz. Average-reference was performed after removing EEG channels in which artifacts affected most of the recording. Computation of the power spectral density was conducted on six-second epochs by means of a Welch’s averaged modified periodogram with a Hamming window. The epochs were divided in 8 segments with 50% overlapping using the built-in MATLAB function pwelch. A threshold based on the mean power at either a low (1–4 Hz) or high (20–30 Hz) frequency band was used to detect and remove artefactual six-second epochs from NREM sleep data [4,25,26]. Artifacts (i.e., muscle twitches, eye movements, heartbeats) were detected and removed from REM data by using independent component analysis (ICA) [27]. Power spectral density was computed on REM sleep data after removal of tonic and phasic REM epochs contaminated with artifacts. After excluding the electrodes located on the neck/face region, we adopted an unbiased approach to the analysis of the frequency power spectrum on the remaining 185 channels, integrating across 1-Hz non-overlapping frequency bins ranging from 1 to 40 Hz. Sleep cycles were defined according to the modified criteria [28] of Feinberg and Floyd [29].

### Statistics

Differences in self-report variables between meditators and controls were investigated with a univariate, repeated-measures ANOVA. As for the socioeconomic status, the within factor had 9 levels (marital status, number of children, and socioeconomic status items: total family income, total family income divided by number of dependents, Hollingshead Index of Social Position, Hollingshead two factor index of social position, how much individual values $100, how much individual values money in general, highest level of education completed, partner's highest level of education completed). For all tests, the p-value threshold was set at 0.05.

Differences in sleep architecture across conditions organized in a 3-way factorial design were investigated with a univariate, repeated-measures ANOVA. The *condition* factor had 3 levels (baseline, Vipassana, and Metta sessions). The sleep *cycle* factor had 3 levels (first, second, and third cycle). The sleep *stage* factor had 4 levels (N1, N2, N3, and REM). Total sleep time (TST) and wake after sleep onset (WASO) were entered in a separate ANOVA that included the *condition* factor and the *sleep parameter* factor (TST and WASO). For both ANOVA designs, the between group factor had two levels, long-term meditators and meditation-naïve controls. Greenhouse-Geisser correction was applied to p-value and degrees of freedom in case of violation of the sphericity assumption.

Differences in topographical NREM and REM sleep hdEEG power between conditions were assessed with statistical non-parametric mapping (SnPM), a permutation approach that accounts for the multiple comparison problem inherent in the analysis of functional neuroimaging datasets [30]. Specifically we adopted a suprathreshold cluster method that quantifies the size of connected regions (clusters) on the thresholded statistical images derived from the observed data and from randomly shuffling the observations between conditions. In this method, the size of each suprathreshold cluster in the observed data is compared against the permutation distribution of the maximal suprathreshold cluster size to obtain the corrected p-value [30]. Specifically, 50000 unique combinations were run for each comparison in order to approximate the actual cluster distribution. The primary threshold t-value was set to 2.069, corresponding to α = 0.05 for the given degrees of freedom. We implemented our in-house MATLAB-based SnPM software in order to control at once for the multiple comparisons deriving from testing numerous scalp channels (185 electrodes), frequency bins (39), and time points (the first 3 sleep cycles). Connected regions were computed in 3 dimensions, namely space, frequency, and time, by letting the algorithm search suprathreshold neighbors not only in the adjacent channels, but also in the adjacent frequency bins and sleep cycles.

In order to control for the effect of time and adaptation to the lab environment, we compared with SnPM the whole night recordings of age and gender matched control participants acquired at 3 different time points, corresponding to the baseline and the two days of meditation practice. Moreover, since we expected a potential effect of adaptation to the lab environment to become evident following the first time point, we compared the average changes in long-term practitioners after days of practice relative to baseline to the respective changes across time in naïve participants.

We used SnPM to investigate the effect of lifetime meditation experience on the changes in spontaneous brain activity during sleep after an intense day of meditation practice over scalp channels, frequency bins, and sleep cycles.

To increase sensitivity, we reduced the frequency space by integrating the frequencies above 15 Hz across 5-Hz bins while keeping a 1-Hz resolution for traditional sleep rhythms [28]. We also separately assessed the contribution of the time spent in the specific meditation style in which the participants were engaged during the day of practice (OM, FA, and LK). In order to control for the influence of outliers on the correlation analyses, we ran the permutation statistics without including data points above or below 1.5 interquartile ranges from the median of practice hours across participants. This criterion yielded to the exclusion of two outliers for lifetime meditation experience, as well as for FA and LK. The primary threshold t-value was set to 2.088, corresponding to α = 0.05 for the given degrees of freedom. The same threshold was set for OM.

## Results

### Socioeconomic status and mental health

The two groups did not differ as for their socioeconomic status [18] (*group* factor, F_1,45_ = 0.1209, p = 0.73; interaction group*questionnaire items, F_2,96_ = 1.457, p = 0.24) and mental health (Quick Inventory of Depressive Symptoms [19], *group* factor, F_1,46_ = 1.0264, p = 0.32; interaction group*QIDS items, F_4,200_ = 0.8735, p = 0.49; Symptom Checklist-90-Revised [20], *group* factor, F_1,46_ = 0.0862, p = 0.77; interaction group*SCL-90 items, F_3,150_ = 0.6662, p = 0.59).

### Sleep architecture

The TST and WASO did not differ between meditators and controls and were not different across conditions (Tables 1 and 2A). An univariate, repeated-measures ANOVA showed a similar sleep architecture (length of cycles and stages) in both groups across conditions, as revealed by the absence of a significant *condition* factor or significant two-way and three-way interactions (Tables 1 and 2B). Thus, the days of meditation did not significantly impact the sleep architecture compared to controls.

**Table 1:** Practitioners’ sleep structure parameters. Times (average across participants ± standard deviation) are expressed in minutes.

	Baseline	Mindfulness	Compassion
Total Sleep Time	365.8±49.8	374.1±50.3	386.8±49.9
Wake After Sleep Onset	73.8±45.6	62.6±43.7	59.3±31.3
**Cycle 1**			
NREM N1	10.8±9.3	6.2±3.8	8.5±5.9
NREM N2	53.5±15.3	45.1±16.6	51.7±26.9
NREM N3	18.6±15.5	15.7±15.4	15.7±16.0
REM	11.6±11.1	12.6±7.5	12.6±8.7
**Cycle 2**			
NREM N1	7.0±4.6	5.6±4.1	8.3±6.1
NREM N2	54.5±14.6	54.6±21.2	59.8±20.0
NREM N3	14.5±16.9	11.8±10.2	10.1±10.0
REM	16.4±13.2	22.4±14.5	18.4±11.3
**Cycle 3**			
NREM N1	6.4±5.1	5.7±3.8	7.5±4.4
NREM N2	54.8±18.3	61.2±12.9	58.6±21.4
NREM N3	5.9±10.5	2.9±4.4	3.2±4.3
REM	20.8±12.9	22.7±13.7	17.3±13.4

**Table 2:** Univariate, repeated-measures ANOVAs showed that sleep structure parameters did not change across conditions. Greenhouse-Geisser correction has been applied to p values and degrees of freedom.

**A**.		
Between factor		
*group* (meditators and controls)	F_1,46_ = 0.0868	p = 0.7696
Within factors		
*condition* (baseline, mindfulness, compassion)	F_2,92_ = 0.4483	p = 0.6383
Interactions		
group*condition	F_2,90_ = 0.0793	p = 0.9224
condition*parameter (TST, WASO)	F_2,46_ = 3.6290	p = 0.0389
group*condition*parameter (TST, WASO)	F_2,46_ = 0.0424	p = 0.9354
**B**.		
Between factor		
*group* (meditators and controls)	F_1,46_ = 1.7333	p = 0.1945
Within factors		
*condition* (baseline, mindfulness, compassion)	F_2,90_ = 0.8301	p = 0.4372
*cycle* (first, second, and third sleep cycle)	F_2,77_ = 4.8454	p = 0.0148
*stage* (N1, N2, N3, and REM)	F_2,92_ = 467.8626	p = 0.0000
Interactions		
group*condition	F_2,90_ = 0.0793	p = 0.9207
condition*cycle	F_3,156_ = 0.8824	p = 0.4624
condition*stage	F_3,148_ = 0.7658	p = 0.5229
group*condition*cycle	F_3,156_ = 0.8498	p = 0.4805
group*condition*stage	F_3,148_ = 1.6188	p = 0.1843
condition*cycle*stage	F_7,327_ = 1.0567	p = 0.3916
group*condition*cycle*stage	F_7,327_ = 0.8710	p = 0.5309

### Lifetime meditation practice

The total amount of lifetime hours of practice ranged from 1,526 to 32,349 hours. The average across the 24 practitioners included in this work was 9,136 hours ± 7,461, SD. The specific contribution of meditation styles to their training were expressed in terms of percentage over the total of daily sitting meditation practice (3,890 hours ± 2,351, SD). Specifically, participants were highly proficient in OM (51.2% ± 23.5, SD), followed by FA (32.9% ± 22.7, SD) and LK (14.5% ± 12.5, SD).

### Vipassana and Metta meditation equally increase low-frequency activity in subsequent NREM sleep

**Fig 1:**
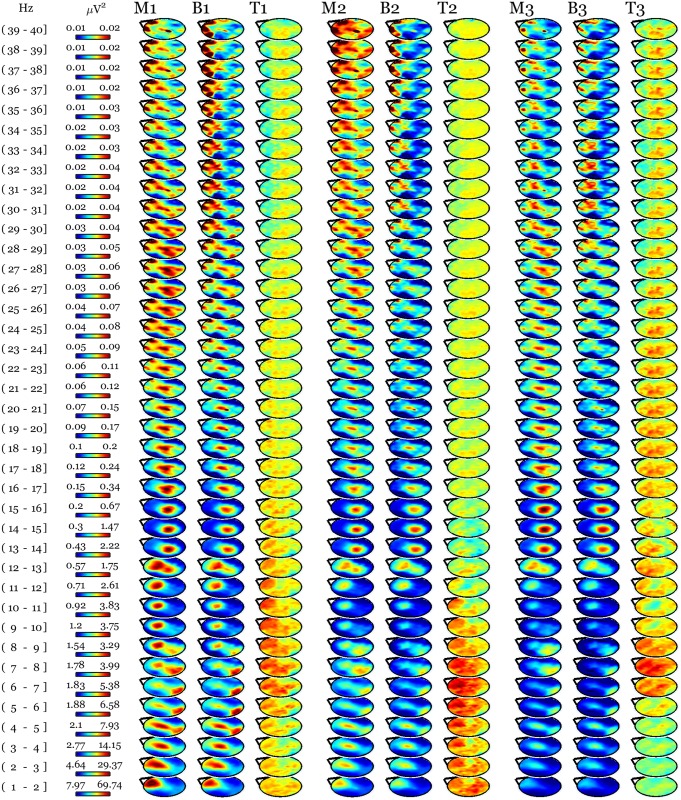
8-h of intense mindfulness and compassion meditation induced an increase in prefrontal and left parietal low-frequency activity (1–12 Hz) in long-term practitioners that extended to high frequencies (25–40Hz) at the end of the sleep night. Average NREM sleep scalp topographies across cycles at baseline (B) and following a daylong meditation session (M) in mindfulness and compassion practice styles (pooled). The first 3 sleep cycles are indexed as 1, 2, and 3. For each cycle, topographical maps of t-values (T) are plotted in the same [-5 5] scale across frequency bins and cycles.

**Fig 2:**
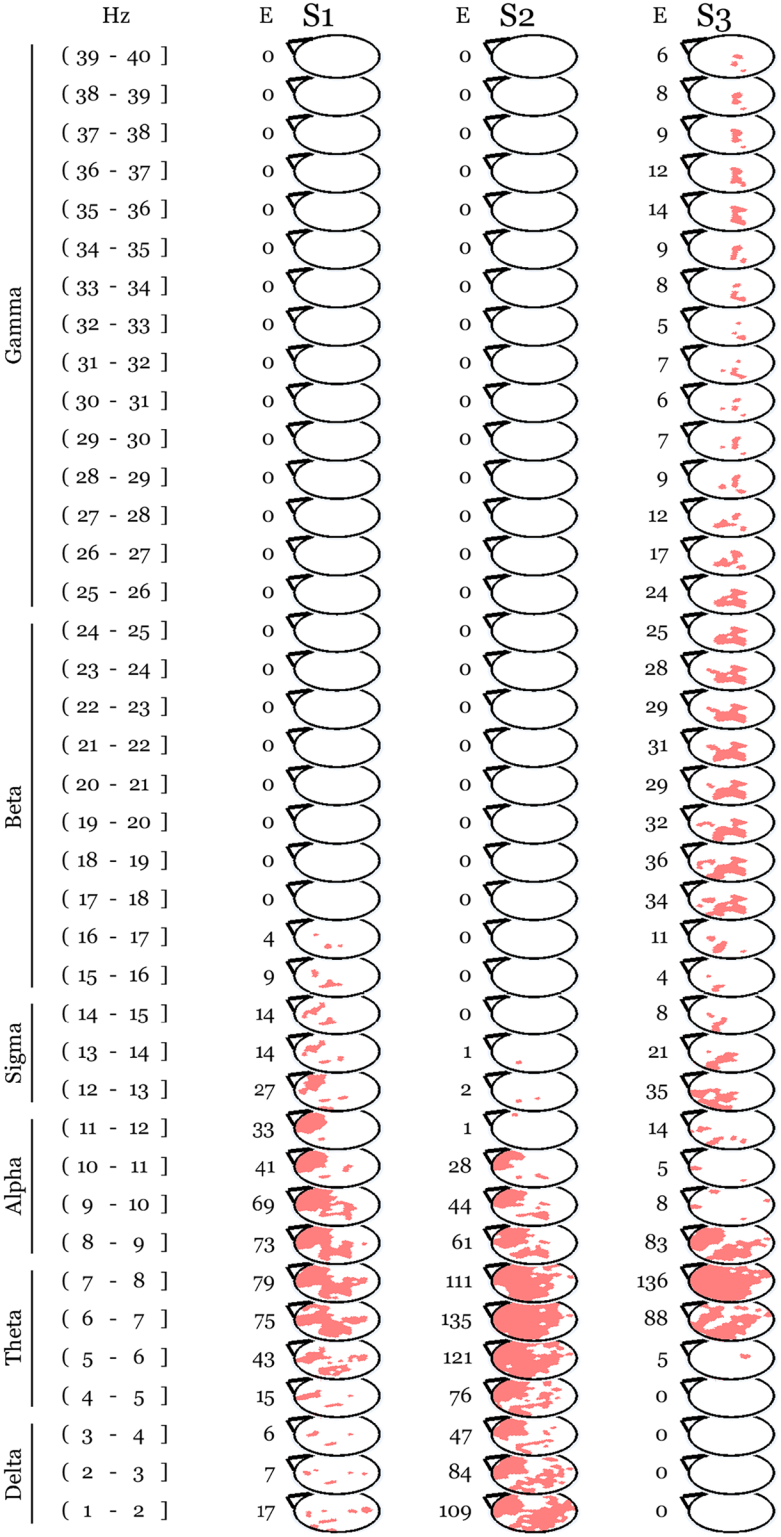
The meditation related increase depicted in Fig 1 survived correction for the multiple comparisons ensuing from testing 185 electrodes, 39 frequency bins, and 3 sleep cycles. The significant cluster (N = 2161, p = 0.046) is shown in pink over white topographical maps (Statistical non Parametric Mapping, SnPM). E indicates the number of significant electrodes for each sleep cycle and frequency bin. S stands for statistical map. The first 3 sleep cycles are indexed as 1, 2, and 3. Traditional frequency bands [45] are reported on the left.

Whole night NREM sleep EEG absolute power values were compared between conditions in 1-Hz bins across a range spanning the interval just above 1 Hz to 40 Hz. Since we did not find any difference in sleep night scalp power following Vipassana and Metta meditation practices, we averaged the intervention conditions together for the purpose of comparing against baseline. This analysis revealed a topographically localized, frequency specific, and time modulated increase in EEG power after a day of meditation practice in Vipassana and Metta meditation as compared to baseline (Fig 1). Suprathreshold cluster analysis confirmed the presence of a prefrontal and left parietal cluster (N = 2161, p = 0.046) spanning low frequencies and spindles in the first cycle with maximal spatial extension in the 8 Hz bin. This activation was prevalent in lower frequency bins in the second cycle where we found the peak in slow wave activity. In the third cycle the frequencies below 5 Hz were not involved, the cluster had its maximal extension in the 8 Hz bin and involved high frequencies up to 40 Hz. Interestingly, this high-frequency effect was localized in a parietal-occipital region, where we recently found a significant increase in gamma power at baseline in long-term practitioners as compared to meditation naïve individuals [4] (Fig 2).

**Fig 3:**
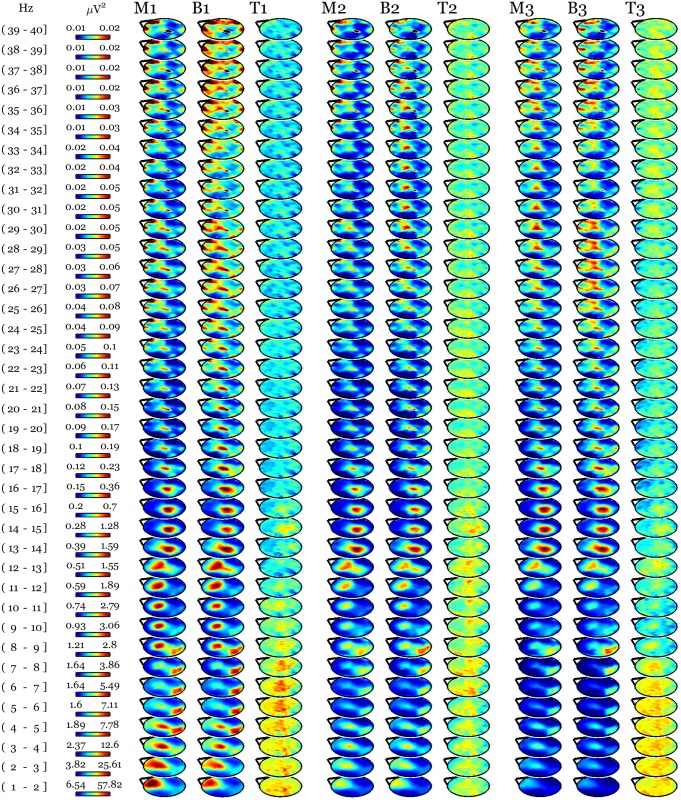
Meditation-naïve individuals recorded at the same time points as practitioners to control for aspecific effect of adaptation to the lab environment did not show changes in scalp EEG between sessions. Average NREM sleep scalp topographies across cycles in control participants at the time points corresponding to baseline (B) and meditation sessions (M, pooled) for practitioners. The naïve individuals did not undergo day of practice. The first 3 sleep cycles are indexed as 1, 2, and 3. For each cycle, topographical maps of t-values (T) are plotted in the same [-5 5] scale across frequency bins and cycles. SnPM statistics confirmed the absence of changes between time points.

**Fig 4:**
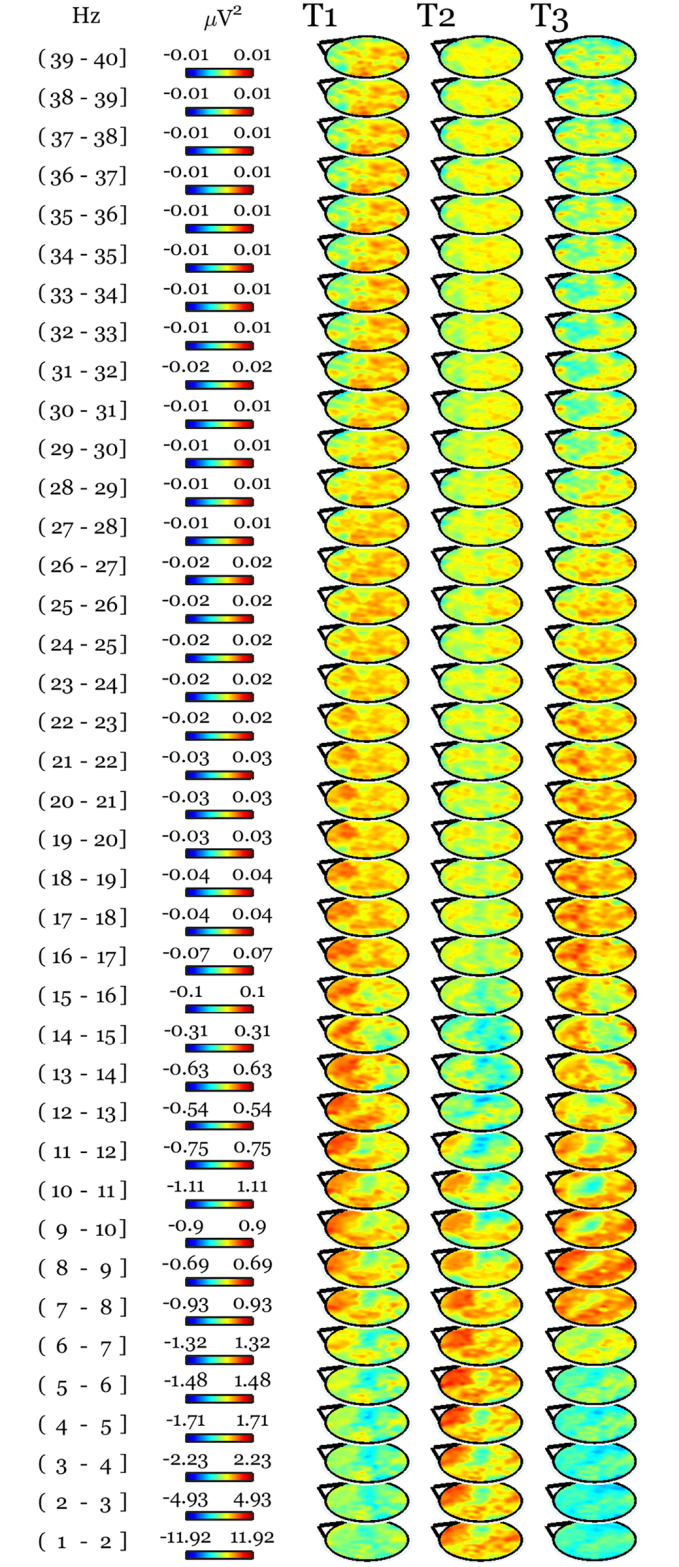
The between groups comparison of the changes relative to baseline shows a pattern similar to the meditation-related changes depicted in Fig 1. For each of the first 3 sleep cycles (indexed as 1, 2, and 3), topographical maps of t-values (T) deriving from the comparison between baseline subtracted meditation sessions (pooled) data in practitioners and corresponding time points in meditation-naïve individuals are plotted in the same [-5 5] scale across frequency bins and cycles. SnPM statistics showed a cluster largely overlapping the one in Fig 2 at a trend level (N = 1929, p = 0.057).

No session effect was found by comparing whole night recordings at the same time points in age and gender matched control subjects (Fig 3). The between groups comparison of the changes relative to baseline still revealed a prefrontal and left parietal increase in low-frequency activity in long-term practitioners after days of practice compared to the same time points in controls (Fig 4), though this cluster did not survive our stringent correction for multiple comparisons (N = 1929, p = 0.057).

### The increase in low-frequency activity correlated with meditation training

**Fig 5:**
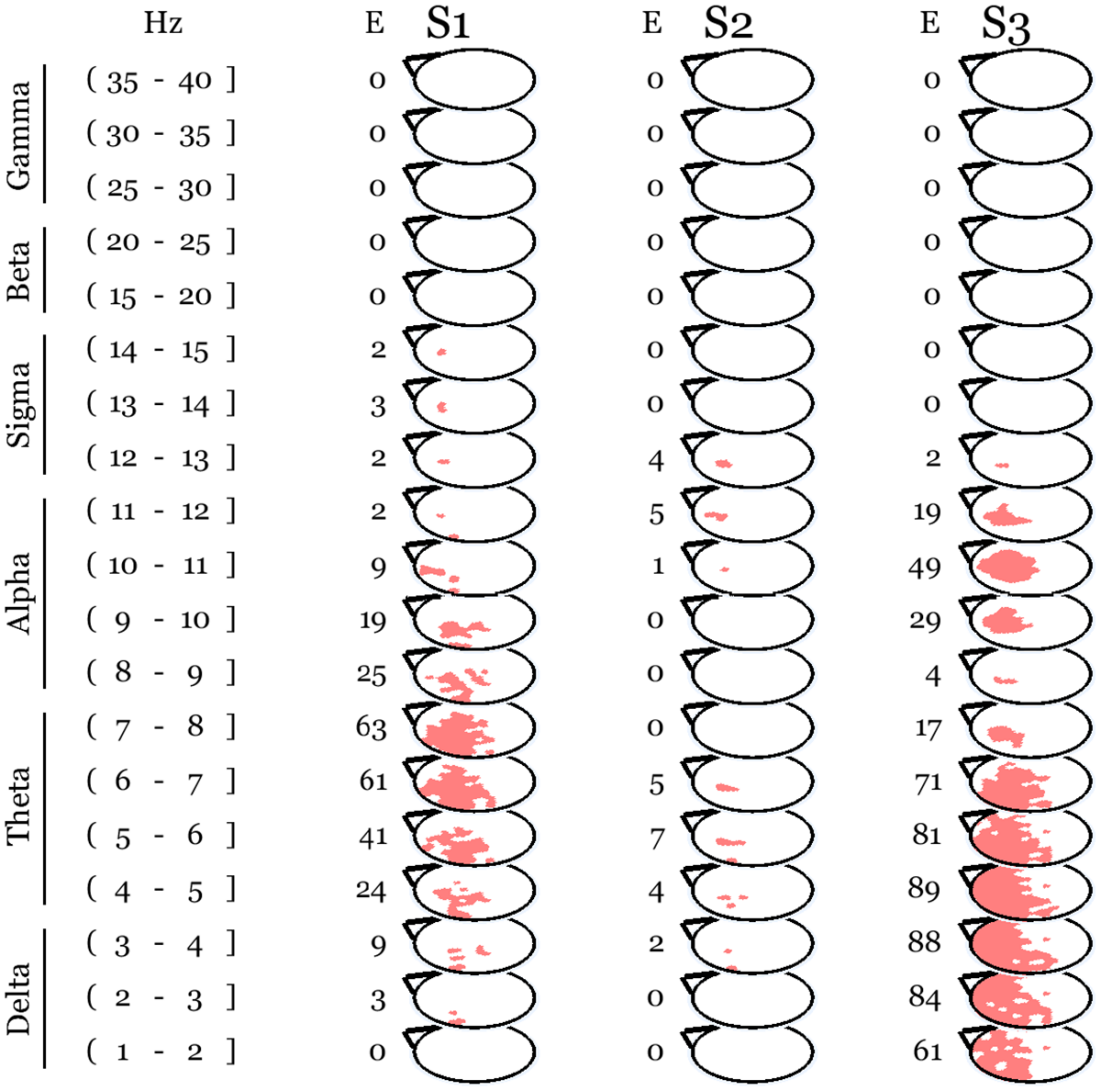
Lifetime open monitoring meditation experience correlated with the topography-specific changes in low-frequency activity following intense daylong meditation practice. The significant cluster (N = 885, p = 0.048) is shown in pink over white topographical maps (Statistical non Parametric Mapping, SnPM). E indicates the number of significant electrodes for each sleep cycle and frequency bin. S stands for statistical map. The first 3 sleep cycles are indexed as 1, 2, and 3. Traditional frequency bands [45] are reported on the left.

We next investigated whether meditation experience could predict the significant changes after intense meditation practice. We found that lifetime meditation practice in open monitoring correlated with the increase predominantly involving theta-alpha power over prefrontal and left parietal electrodes during the sleep night following the intense meditation sessions (suprathreshold cluster analysis, N = 885, p = 0.048). The overlapping with the changes in low-frequency activity after intense meditation practice (Fig 2) was more pronounced in the first cycle (Fig 5).

### REM sleep EEG power was not affected by the intensive meditation sessions

After computing the spatial distribution of power normalized to the mean power across all electrodes, we also performed topographic EEG analysis of REM sleep. As previous literature has suggested functional differences between tonic and phasic REM, we separated these two REM sleep patterns in our analysis [31–33]. SNPM analyses revealed no topographical differences in any frequency bins nor sleep cycles in REM sleep between intervention conditions and baseline.

## Discussion

In this study we found a significant increase in scalp EEG power predominantly localized in low frequency activities (1–12 Hz) across sleep cycles after an intense 8-hour session of mindfulness meditation (Vipassana) and compassion meditation (Metta) practice, which positively correlated with the lifetime meditation experience. This increase was localized over prefrontal and parietal electrodes for both styles of meditations and was specific for NREM sleep. This effect peaked at the transition between the alpha and theta bands (8 Hz) during the first sleep cycle, whereas during the second cycle it extended to slow wave activity (SWA) and spread across the scalp surface involving also posterior regions.

A localized increase in low-frequency (≤ 11Hz) sleep EEG activity has been shown to be induced by specific forms of training [6–8]. Furthermore, the recovery sleep following ≥24-h of prolonged wake while listening to a story or performing a visuomotor task was characterized by experience-induced changes in low-frequency activity, indicating a process-specificity of these frequency bands. The task-specificity of this effect was reflected by higher low-frequency changes occurring in task-related scalp regions during sleep, which were correlated with changes in theta power during wakefulness in the same locations [7]. Here we found a localized increase in low-frequency activity, including theta and alpha oscillatory activities at the beginning of the night that during the second cycle involved also SWA. This delayed increase in SWA is consistent with the plastic changes induced by waking experiences not immediately preceding sleep [8]. Specifically, we recently showed that learning a visuomotor task in the morning, and then engaging in the usual daily activities, was followed by a region-specific increase in sleep SWA during the night, which peaked during the second sleep cycle. Consistently, the process-specific increase in low-frequency activity resulting from intense meditation practice shown here followed a similar temporal pattern, while localizing in different scalp regions specific to the meditation training. The relationship between this localized increase in low-frequency activity and meditation training was highlighted by an increase in sleep spindle activity (sigma frequency band, known to be locally modulated by training [34]) in the same region early in the night.

The increase in alpha-theta power was not related to changes in self-reported sleepiness and was accompanied by increase in delta power compared to the baseline, thus suggesting that these findings are not due to a decrease in sleep depth. While alpha EEG during sleep is most frequently considered as a sign of arousal [35] or covert waking activity [36], and some forms of arousals during sleep are accompanied by so-called delta-injections [35], we had found in our previous work that meditators and naïve individuals did not differ in terms of their number of microarousals [4]. However, the previous study did show that long-term meditators differed in their gamma band activity relative to controls suggesting a specific meditation-related increase. Moreover, under the point of view of the macroscopic sleep structure, the wake after sleep onset did not differ at any time point between meditators and controls. It needs to be underscored as well that the localization of the increase does not match the topography of the alpha increase associated with perception of poor sleep quality, and delta activity is generally associated with sleep stability [37] further suggesting a different, meditation-related, functional significance of our finding. Likewise we can exclude that our findings were partially mediated by the cognitive demand of the Go/No-go task, given that this attentional task was administered at baseline as well as after the meditation sessions. An increase in EEG alpha-theta activity has also been reported during deep, slow wave sleep in transcendental meditation practitioners, a mantra-based meditation [38].

A prefrontal alpha-theta increase has been associated during wakefulness with different styles of meditative practices, including mindfulness-related practices. In addition, Vipassana meditation, has also been linked to state-related increase in posterior gamma power [1,39], and Metta meditation has been found to induce a sustained increase in gamma power over a large number of scalp electrodes [2]. We recently found the signature of these meditation-related effects in the baseline sleep of our practitioners, as an increase in parietal-occipital gamma power in NREM sleep [4]. The intensive day of practice investigated here induced a further measurable increase in gamma power that was found in the third sleep cycle following the meditation sessions. This change occurred in a parietal region overlapping the one found significant at baseline [4]. Our results point to a different involvement of prefrontal-parietal low-frequency EEG activity and parietal-occipital gamma power in mediating the acute and long-lasting effects of meditation on sleep EEG activity respectively.

Consistent with our previous finding of a positive correlation between sleep parietal-occipital gamma activity and the length of the lifetime daily meditation practice [4], the acute prefrontal and parietal changes predominant in low-frequency activity during sleep in the same experienced meditators correlated with their level of experience. More specifically, the time spent in open monitoring meditation, the most common form of training inside the Vipassana tradition, predicted the increase in low-frequency activity after the days of practice. This correlation points to a reactivation of neural circuits involved in attentional processing sustaining both Vipassana and Metta meditation practices. We cannot however exclude that the loving-kindness meditation could induce changes in the activity of brain regions not accessible to be studied through EEG. Source modeling and connectivity analyses will help highlight domain-specific effects and clarify the underlying neural mechanisms.

At this stage, we can only speculate about the functional significance of our findings. One possibility is that prefrontal-parietal alpha-theta EEG activity could represent a persisting activation of underlying neuronal circuits engaged by the intense meditation practice during the day [39]. Those circuits could be still involved in meditation related activities, or be reactivated during sleep, or be more responsive to internal/external stimuli. We speculate that these changes could be related to the generation of mental representations during sleep out of memory traces [40]. An intriguing future research question will be to investigate whether these changes in low-frequencies are linked to an enhancement of the awareness of internal and external stimuli during NREM sleep [3] with increased top-down control of the contents of conscious experience [41]. An increase in theta and alpha oscillations coexisting with slow wave activity has been previously described in transcendental meditation practitioners and is paralleled with the phenomenon called witnessing of sleep [38]. Moreover, an increase in gamma activity has been associated with lucid dreaming [42]. These observations, together with the dependence of the changes in prefrontal-parietal alpha-theta and parietal-occipital gamma oscillations on meditation experience, suggest a possible increase in the level of awareness [43,44] in meditation practitioners not only during waking activities, but also during sleep.

In conclusion, we were able to show the acute effects of meditation training during subsequent sleep. Future work is needed to elucidate the functional significance of these changes. Specifically, the low-frequency oscillatory activities increase observed over prefrontal and left-parietal electrodes following an intensive day of meditation practice could index alpha mediated top-down inputs [41] regulating the parietal-occipital regions, which show long-lasting plastic changes in long-term meditators. Those plastic changes would in turn sustain enhanced gamma mediated conscious experience during sleep. The alpha-gamma interplay is particularly interesting in light of the role of these frequency bands in mediating top-down and bottom-up brain connectivity respectively [41] and suggests that training voluntarily directed attention creates in the long run effortless enhanced cognitive abilities mediated by bottom-up gamma processing [10]. The consolidation of these plastic changes could be mediated by the posterior spreading of the cortical activation with significant increase of the slow wave activity during the second cycle.
